# Untitled public forestlands threaten Amazon conservation

**DOI:** 10.1038/s41467-023-36427-x

**Published:** 2023-03-01

**Authors:** Paulo Moutinho, Claudia Azevedo-Ramos

**Affiliations:** 1 Amazon Environmental Research Institute – IPAM Amazon, Brasília, DF Brazil; 2 Center for Advanced Amazonian Studies – NAEA, Federal University of Pará, Belém, PA Brazil

**Keywords:** Conservation biology, Environmental impact, Politics

## Abstract

A large proportion of recent Brazilian Amazon deforestation is occurring on untitled public forestlands through land grabbing. This emerging risk demands long-term conservation strategies. Here we propose prioritizing land tenure security, technological improvement, and law enforcement.

The deforestation rate in the Brazilian Amazon is growing rapidly again after being temporarily brought under control and reduced by 80% between 2005 and 2012^1^. Deforestation in the region has increased from 4600 km^2^ in 2012 to 13,000 km^2^ in 2021^2^, driven by land grabbing on public land and conversion of forest to agriculture (e.g., soybeans) and pasture on private land.

Land-tenure insecurity has been a long-lasting issue in the Brazilian Amazon^3^. It involves the uncertainty of recognition of a person’s right to land (or a public body’s management right) and the consequent risk of having it threatened and even lost by competing claims. Land-tenure insecurity, thus, is at the root of the difficulties in adopting sustainable land use models.

Building on recent findings from Pacheco and Mayer^4^ and past experiences, here we discuss how to curb deforestation and preserve large tracts of Amazon untitled public forest land using durable strategies. The unfolding of these strategies into effective and concrete practices will need collaboration of multiple academic and non-academic stakeholders.

## Untitled Amazon forestlands as a conservation risk

**Fig. 1:**
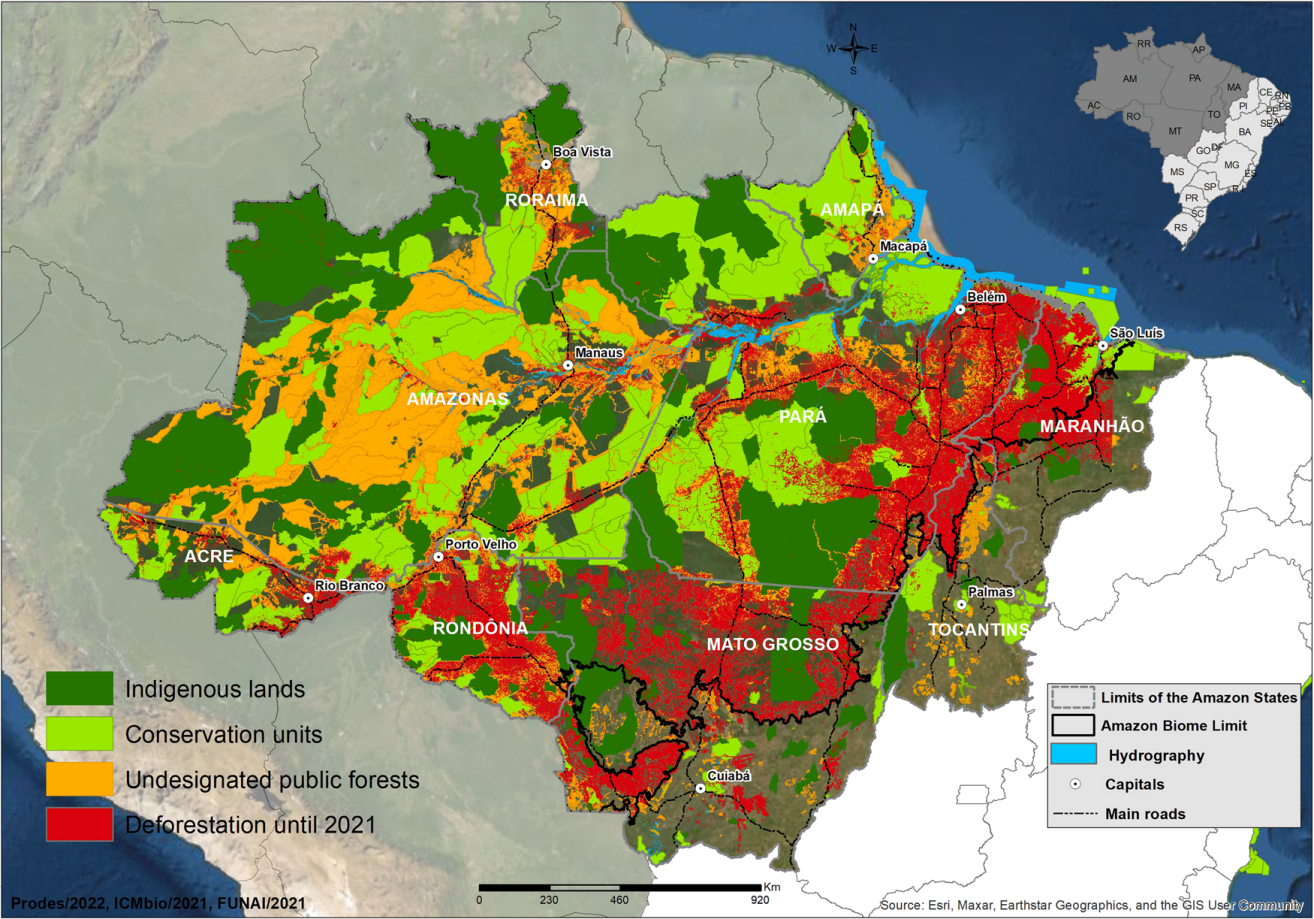
Protected areas, undesignated public forests, and the deforestation in the Brazilian Amazon. Undesignated public forests (in orange), conservation units (in light green), indigenous lands (in dark green), and deforestation by 2021 (red dots). Source: refs. ^2,21–23^.

Around 50% of the Brazilian Amazon deforestation occurs on public land^5^, particularly in the so-called undesignated public forests (UPFs)^6,7^. The UPFs (Fig. 1) are untitled lands that do not belong to any tenure category specified by law^6,7^. These public forests cover an area of 56 million hectares (the size of Spain) and hold a stock of seven billion tons of carbon^7^—almost 1 year of global emissions. With poorly defined tenure rights, UPFs have been an easy target for land grabbers and illegal natural resource exploitation.

The study by Pacheco and Mayer^4^ addresses a critical environmental issue for land use in Brazil—how do land tenure regimes (e.g., private land, protected areas, Indigenous land) affect deforestation? The authors analyzed 33 years (between 1985 and 2018) of agriculture-driven deforestation across Brazilian forestlands. Their main conclusion is that any formal land tenure regime (even private ownership) reduces deforestation compared to undesignated public lands. Reinforcing their results, from 2019 to 2021, around 30% of the annual deforestation in the Brazilian Amazon occurred in UPFs^6^^,^^7^. And yet, neither the Brazilian states in the Amazon region nor the Federal government is fulfilling their legal duty to categorize the UPFs. Consequently, this large area of dense and pristine forest is vulnerable to land-grabbing and illegal deforestation.

Pacheco and Mayer^4^ argue that changes in the status of protected areas or Indigenous lands, through privatization, for example, would increase deforestation. As already indicated by other studies, conservation areas and Indigenous land have proven effective in halting the advance of deforestation in several regions^1,8^. For instance, the significant decrease in Amazon deforestation between 2005 and 2012 can be partly explained by the designation of 24 million hectares of public land for protected areas, including Indigenous lands (which are formally part of Brazil’s protected area system)^1^. The threats on the status of Brazilian protected area by the ongoing process of protected area downgrading, downsizing, and deregulating is already a reality^9^, increasing the complexity of the Amazon conservation puzzle.

Therefore, actions are needed to increase protection of large tracts of forests by preventing further deforestation in UPFs and strengthening Amazon rainforest conservation in the long term. Those actions should be strong enough to withstand periods of political antagonism to forest protection, as seen between 2018 and 2022. Next, we focus on how to curb the advance of land grabbing of untitled Amazon forestlands, which have faced greater deforestation pressure since 2015.

## The way forward

There is a recent change in the modus operandi of Brazilian Amazon deforestation. The proportion of illegal deforestation in public land increased from ~43–44% (2015–2018) to ~49–52% (2019–2021)^10^. Land grabbers occupy public lands (deforesting or raising cattle) in a high-risk expectation of receiving title to the land and/or trading the land with significant returns (land speculation)^6^^,^^7^. Therefore, we argue that it is crucial to rapidly assign most of the Amazon’s UPFs to land tenure regimes associated with conservation. Land-tenure security will bring greater governance and protection to these areas. Achieving this goal requires a combination of three measures: (1) careful attention to the choice of land tenure categories for UPFs, (2) technological improvements, and (3) law enforcement.

### Choice of land tenure category for UPFs

Public lands in Brazil include several categories, such as conservation areas (with several subcategories under law number 9985/2000), Indigenous lands, and rural settlements, among others. Therefore, the category choice for each undesignated public land area requires studies to determine those lands’ social, environmental, or productive suitability, taking note of their histories of occupation, cultural importance, and potential uses. The unpopulated forest is a myth. Most of the areas in the Amazon have been occupied by human populations—traditional communities, indigenous villages, uncontacted tribes, “riverside” (*ribeirinho*) peoples, or small farmers—for generations. Ancestral occupation of land without proof or associated studies, however, does not guarantee land rights. Therefore, to avoid unfair competition for land and unilateral political decisions, the best choice of land category for a given UPF to meet social, ecological and economic demands would benefit from active social participation, multidisciplinary scientific studies, in situ observations, and innovative technologies (e.g., remote sensing, data processing capabilities, machine learning, cloud computing) to provide fast, scalable, and quality information.

Final allocation decisions, however, must be preceded by participatory and transparent consultation processes to avoid conflicts and safeguard land rights. The measure of assigning tenure categories to the UPFs has a high level of complexity in itself and may benefit from the support of multi-actors (e.g., governments, academia, civil society, private sector) at multi-levels (e.g., studies, participation processes, decision-making processes) and multi-scales (local, regional and national). Despite the complexity, there are examples in the early 2000s of joint efforts to allocate land (“Terra Legal” Program) and create protected areas on a large scale and in a short period of time in the Brazilian Amazon. We emphasize, however, that the tenure categories selected for the UPFs need to maintain forest cover, remain in the public domain in compliance with national laws, and enhance long-term Amazon conservation, respecting the rights of resident populations.

### Technological improvements to control land grabbing in UPF

Lasting conservation of the Amazon rainforest depends on ending land-grabbing and illegal deforestation in public forests (designated or undesignated). However, land grabbers are using a self-declaratory tool to declare illegally invaded public lands as private properties, which demands immediate technological improvements to the system.

The Rural Environmental Registry (CAR is the Portuguese acronym) is a mechanism of environmental oversight of private lands under the Brazilian Forest Code (Law 12,651/2012). CARs are registered on a web-based platform (Rural Environmental Registry System – SICAR). By law, landowners must self-declare their property boundaries and land use types (e.g., residential, agricultural, protection) in SICAR, respecting legally required protection of certain forest areas and watercourses. Then, a state environmental agency must validate the information. Unfortunately, the validation process has been extremely slow (e.g., <1% of 6.6 million properties validated by 2022)^11^, creating opportunities for fraud.

A priority action for improving the system, thus, is to speed up the CAR validation process, eliminating illegal registrations from the system.

The validation task would be much more simplified if the federal and state governments had an integrated CAR database and system. Brazil has unique and comprehensive data on land tenure (e.g.,^12^), land use changes^13^, and public forests (Brazilian Forest Service), among many others. The challenge lies in integrating these data, which would allow quick consultation and visualization of the areas, compliance verification, record validation, and facilitation of the decision-making process. The integrated system would provide powerful tools in the fight against illicit activity without penalizing those in compliance with the regulations. This is a straightforward task that can be accomplished quickly if governments, academics, and technical experts collaborate in solving technology and data management challenges associated with systems integration.

Removing the illegal CAR from the SICAR database is crucial. Around 20 million hectares (20%) of UPFs are already illegally declared as private property^7^. Approximately 3.4 million hectares of UPFs that were illegally deforested by 2020, about 65%, overlapped with illegal CAR^7^. This scenario represents a time bomb of deforestation, ready to be detonated over the coming years. Land grabbers have also been using false property registration to receive economic advantages, such as bank loans. A Resolution (No. 3545/2008) of the Central Bank of Brazil requires evidence of environmental compliance (such as CAR) from producers to qualify for public loans. These loans, in turn, when granted to land grabbers, may be inadvertently funding new deforestation in public areas.

### Law enforcement

Among different ways to prevent and discourage crimes, punishment is a measure usually applied for law violations. Fines, for instance, are commonly used in environmental crimes. Uncollected fines, thus, signal impunity and motivate the perpetuation of environmental crimes^14^. In Brazil, gaps in regulations^15^ provide opportunities for noncompliance with fines, which often expire after successive judicial appeals. Therefore, those who invade and deforest public forests have invariably escaped the rigors of the law. In the current judicial system, other sanctions, such as embargoes on illegally deforested rural properties and confiscation of means of production, proved to be more effective^14^.

Administrative and regulatory reforms, thus, are needed so that offenders can be prosecuted promptly, receive sanctions in proportion to the severity of their violations, and be effectively and quickly punished. These reforms will require experts in law and administrative processes to find the best way to solve gaps, but the measure’s success depends on support from within the regulatory/supervisory agencies and in the legislative bodies favorable to more robust and reliably enforced rules. For instance, a point worth considering is that although cumulative and financially heavier environmental penalties may influence would-be offenders^16^, the deterrent effect of an increase in enforced penalties (e.g., with a credible collection of fines, embargoes on properties, among others) may prove greater than highly severe, but difficult to enforce, sanctions. While still to be tested in environmental crimes, the assumption is that, pedagogically, some “swift and certain” punishment is preferable to impunity due to ineffective or too harsh legal coercion^17^.

## Final remarks

Adding to Pacheco and Meyer’s^4^ results that for reducing deforestation any formal land tenure regime is better than untitled lands, here we have recommended priority actions for structural interventions in a complex scenario to stop deforestation in UPFs and buy time to set in motion many other long-lasting transformations necessary for effective Amazon conservation. These should ideally be accompanied by alternatives to the development model prevailing in the Brazilian Amazon, which entails the replacement of the forest for other land uses. The deforestation reduction in the Amazon is linked to the promotion of sustainable development based on socioeconomic stability that may alleviate pressure on the forest, as learned from experiences such as community forest management, adding value to forest products, ecosystem services valuation, agricultural intensification and fair trade and purchase For instance, using the “conservation-through-use” approach, there are many examples of self-organized communities in the Amazon contributing to local livelihoods, forest-based development and forest protection through micro-enterprises spread across different tenure types (e.g., production of timber, Brazil nuts oraçai)^18^. Sustainable practices and fair trade by large-scale producers^19^ are also key for Amazon conservation and development due to their potentially large impact.

Reforestation may contribute to minimizing losses of multiple ecological services, but centuries-old forests are difficult to replace. Therefore, it is crucial to focus on strategies that guarantee the durable protection of existing forests, especially considering their role in mitigating global warming. Brazil has a history of successful initiatives^20^ and social engagement (e.g., grassroots movements, coalitions among civil society institutions, coalitions between civil society institutions and private business). These initiatives are providing tools (e.g., Mapbiomas data set), monitoring (e.g., Climate Observatory, Forest Code Observatory, Amazon Trade and Environment Observatory), and multi-stakeholder discussion forums (e.g., Coalition Brazil – Climate, Forests and Agriculture) that help reduce Amazon deforestation.

An opportunity to increase forest protection presents itself after the recent Brazilian presidential election (2022) and announced positive shifts to environmental policies. What is most needed now is a practical demonstration of the apparent political will of the new Central Administration to respond to Brazilian society’s desire, along with that of many other nations, to conserve the Amazon, protecting its people, biodiversity, and the global climate.
