# Author Correction: Untitled public forestlands threaten Amazon conservation

**DOI:** 10.1038/s41467-023-37218-0

**Published:** 2023-03-14

**Authors:** Paulo Moutinho, Claudia Azevedo-Ramos

**Affiliations:** 1 Amazon Environmental Research Institute—IPAM Amazon, Brasília, DF Brazil; 2 Center for Advanced Amazonian Studies—NAEA, Federal University of Pará, Belém, PA Brazil

**Keywords:** Conservation biology, Environmental impact, Politics

Correction to: *Nature Communications* 10.1038/s41467-023-36427-x, published online 1 March 2023

The original version of this Article contained an error in the title, which was previously incorrectly given as ‘Untitled public forestlands threat Amazon conservation’. The correct version states ‘threaten’ in place of ‘threat’. This has been corrected in both the PDF and HTML versions of the Article.

